# Participant Characteristics Associated with Symptomatic Improvement from Yoga for Chronic Low Back Pain

**DOI:** 10.4172/2157-7595.1000151

**Published:** 2014-01-11

**Authors:** Kim M Stein, Janice Weinberg, Karen J Sherman, Chelsey M Lemaster, Robert Saper

**Affiliations:** 1 Boston University School of Medicine, USA; 2 Department of Family Medicine, University of Virginia Medical Center, USA; 3 Department of Biostatistics, Boston University School of Public Health, USA; 4 Group Health Research Institute, Group Health Cooperative, Seattle, WA and Department of Epidemiology, University of Washington, Seattle, WA, USA; 5 Department of Family Medicine, Boston University School of Medicine and Boston Medical Center, USA

**Keywords:** Back pain, Low back pain, Chronic pain, Yoga, Hatha yoga, Socioeconomic status, Alternative medicine, Complementary medicine, Integrative medicine

## Abstract

**Context:**

Studies suggest that yoga is effective for moderate to severe chronic low back pain (cLBP) in diverse predominantly lower socioeconomic status populations. However, little is known about factors associated with benefit from the yoga intervention.

**Objective:**

Identify factors at baseline independently associated with greater efficacy among participants in a study of yoga for cLBP.

**Design:**

From September–December 2011, a 12-week randomized dosing trial was conducted comparing weekly vs. twice-weekly 75-minute hatha yoga classes for 95 predominantly low-income minority adults with nonspecific cLBP. Participant characteristics collected at baseline were used to determine factors beyond treatment assignment (reported in the initial study) that predicted outcome. We used bivariate testing to identify baseline characteristics associated with improvement in function and pain, and included select factors in a multivariate linear regression.

**Setting:**

Recruitment and classes occurred in an academic safety-net hospital and five affiliated community health centers in Boston, Massachusetts.

**Participants:**

Ninety-five adults with nonspecific cLBP, ages ranging from 20–64 (mean 48) years; 72 women and 23 men.

**Outcome measures:**

Primary outcomes were changes in back-related function (modified Roland-Morris Disability Questionnaire, RMDQ; 0–23) and mean low back pain intensity (0–10) in the previous week, from baseline to week 12.

**Results:**

Adjusting for group assignment, baseline RMDQ, age, and gender, foreign nationality and lower baseline SF36 physical component score (PCS) were independently associated with improvement in RMDQ. Greater than high school education level, cLBP less than 1 year, and lower baseline SF36 PCS were independently associated with improvement in pain intensity. Other demographics including race, income, gender, BMI, and use of pain medications were not associated with either outcome.

**Conclusions:**

Poor physical health at baseline is associated with greater improvement from yoga in back-related function and pain. Race, income, and body mass index do not affect the potential for a person with low back pain to experience benefit from yoga.

## Introduction

Low back pain is the most common pain condition in the United States. An estimated 5–10% of adults in the United States experience chronic low back pain (cLBP) [1,2]. Not surprisingly, this is a major driver of costs and use of healthcare resources, accounting for approximately 2–3% of all physician office visits annually [2,3]. cLBP causes significant morbidity and disability in sufferers, which measurably impacts their quality of life [3,4]. A variety of treatments are commonly used: educational interventions, exercise, various classes of oral medication including non-steroidal anti-inflammatory drugs and opiates, spinal and trigger point injections, behavioral therapy, physical therapy, and major surgery [2–5]. Relief from these options is often incomplete, leading many patients to turn to complementary therapies in an attempt to lower pain and improve function [6–8].

Yoga is commonly chosen by some patients with cLBP as an alternative therapy [5,6]. Studies have shown yoga’s effectiveness in reducing pain and improving function in predominantly white middleclass populations [9]. Newer studies suggest that yoga is effective for moderate to severe cLBP in a diverse predominantly lower socioeconomic status population [10,11]. However, like most treatments, some people appear to gain greater benefit from a yoga intervention than others. Little is known about what socioeconomic factors at baseline may predict greater effectiveness. Among participants interested in using yoga for low back pain, potentially identifying any sub-populations that are more likely to benefit would be advantageous to providers, patients and payers. A search of the literature was conducted to identify studies exploring any associations between socioeconomic factors and outcome for yoga and chronic lower back pain. None were identified. To this end, we performed a secondary analysis on data gathered for a yoga dosing study comparing weekly to twice-weekly classes of hatha yoga in a primarily lower socioeconomic urban population. Our goal was to gain a better understanding of who may benefit most from yoga for cLBP to help tailor interventions offered to patients and improve allocation of resources.

## Methods

A comprehensive description of the original study’s methods can be found elsewhere [10]. Briefly, from September-December 2011, a 12-week randomized dosing trial was conducted comparing weekly vs. twice-weekly 75-minute hatha yoga classes for 95 predominantly low-income minority adults with nonspecific cLBP. Recruitment and classes occurred at Boston Medical Center, an academic safety-net hospital, and five affiliated community health centers in Boston, Massachusetts. Participant characteristics were collected at baseline, including sociodemographics, duration and severity of back pain, employment status, health-related quality of life (SF-36), and previous treatments. The original study found improvement in both of its primary outcomes, back-related function (modified Roland-Morris Disability Questionnaire, RMDQ) and low back pain score (LBPS; rated on a 0–10 scale for the previous week) for both groups but no difference between groups [11]. A total of 95 adults with nonspecific cLBP were enrolled, with ages ranging from 20–64 (mean 48). They consisted of 72 women and 23 men. Of these, 79% were U.S. born, with 21% born abroad. Insurance consisted of 56% public insurance (Medicaid, Medicare, Commonwealth Care) and 43% private insurance. The participants’ reported race was 55% black, 18% white and 27% other; 10% identified themselves as Hispanic. Ninety-one individuals returned for follow-up visits, and our analysis was restricted to these individuals. Of these, 89 had complete baseline data and were used in the adjusted analyses.

This study used the collected participant information to determine which factors measured at baseline beyond treatment assignment were associated with change from baseline to Week 12 in either primary outcome – RMDQ and LBPS. For each primary outcome, we calculated a change score by subtracting baseline from 12 week values. We began by using bivariate testing to assess the relationship between a set of a priori baseline factors and change in each primary outcome. T-test, ANOVA, and Pearson correlation were used as appropriate. We then considered all variables with a p-value less than 0.20 on bivariate testing for our multivariate linear regression models, one for each of our primary outcomes. We used a backwards selection modeling strategy. The least significant (highest p-value) variable was iteratively removed until all remaining variables had a p-value of less than 0.10. In addition, an a priori decision was made to include the baseline outcome measurement; age, gender, and treatment assignment in the final model regardless of final p-value for adjustment given the known effect of these variables on many outcomes such as recovery from back pain. The number of missed days of work was found to have a bimodal distribution, and all participants were categorized as having either no missed work or some missed work in the previous 28 days due to their back pain. For all variables, an alpha of 0.05 in the final regression model was used as the cutoff for significance; any variable with a p-value between 0.05 and 0.10 remained in the model as a possible confounder. For removed variables, the adjusted p-value at the time of removal is reported.

To explore the robustness of our results, we categorized individuals post-hoc into improvers and non-improvers, defined as > 30% or <30% change from baseline, respectively. Thirty percent change from baseline in pain or function is often considered a minimal clinically significant change in back pain studies [12]. The same factors used for the final linear regression model were entered into a logistic regression model with improvement as the dependent variable.

## Results

Table 1 shows baseline characteristics of the 95 participants. The unadjusted (bivariate) analyses are reported in Tables 2 and 3 for change in both RMDQ and LBPS. Higher baseline score, and lower SF-36 physical component score (PCS) were associated with greater improvement in both RMDQ and LBPS. Country of birth (foreign born) and missed days of work (fewer) were significantly associated with greater improvement in RMDQ. Education (higher overall level) and time since cLBP onset (more recent) were associated with greater LBPS improvement. Previous back pain treatments were not significantly associated with improvement.

A total of 89 participants had complete data and were included in the adjusted analyses (Table 4). In addition to the baseline outcome measurement, age, gender, and treatment assignment, nationality, primary language, Hispanic ethnicity, education, unemployment, pain-related missed days of work, baseline BMI, and time suffering from cLBP, SF-36 PCS, and previous osteopathic manipulation were all included in the initial model. Foreign nationality and lower baseline SF- 36 PCS were independently associated with improvement in RMDQ. Greater than high school education (22% greater improvement), cLBP less than 1 year duration (12% greater improvement), and lower baseline SF-36 PCS (8% greater improvement for every 1 point change) were independently associated with improvement in LBPS. Use of pain medications, BMI, income, gender and race were not associated with either primary outcome. Robustness of the results was seen across modeling methods, including stepwise modeling. No meaningful differences were seen between the linear regression results reported here and logistic regression results for factors independently associated with > 30% improvement from baseline, modeled using both the same initial factors with backwards selection and no additional selection with only the predictors from the final linear regression model

## Discussion

In a secondary analysis of a randomized dosing trial of yoga for cLBP in a diverse urban population, we found that lower levels of physical health as measured by the SF-36 PCS were predictive of greater improvement in both low back pain intensity and back-related function. Chronic low back pain of shorter duration and college education were both independently associated with improvement in pain, but not function. Interestingly, foreign nationality was an independent predictor of improvement in function. Other sociodemographic factors such as age, gender, race, ethnicity, employment, and income were not independently associated with either outcome of improvement.

Although potential mediators of yoga’s impact on low back pain have been investigated [13], little research has been conducted on whether sociodemographic and clinical factors at study entry are independently associated with improvement. However, this question has been addressed in a number of observational and interventional back pain studies not involving yoga. In a secondary analysis of an acupuncture intervention for back pain, Sherman et al. [13,14] found that “the strongest predictors of improvement in back function and symptoms were higher baseline levels of these measures, receipt of an acupuncture treatment, and non-use of narcotic algesics [14].” The UK BEAM trial interestingly found that while age, work status, highest level of education, pain and disability, quality of life and baseline beliefs all predicted improvement in back dysfunction, they were not significantly associated with receiving specific treatments [15]. Both of these studies examined interaction terms to determine if the baseline characteristics influenced response to the treatment itself, rather than merely predicting improvement alone. In contrast, we attempted to identify which patients improved overall. This reflected our dataset; we had no true control group as the dataset was derived from a study of two different doses of yoga.

In a study of cognitive behavioral therapy for chronic pain patients, McCracken et al similarly did not find that patient sociodemographic variables predicted to outcome [16]. Other studies have also shown that overall age, gender, marital status, and duration of pain are not significantly related to outcome [17–21]. In contrast, level of education has been found to impact outcomes. In a prospective cohort study of cLBP patients, Costa et al found less education was associated with slower improvement [22]. Whereas some studies have found less improvement in patients with high levels of baseline pain and physical dysfunction [22,23], we found that yoga was associated with improvement in individuals with lower levels of physical health as measured by the SF-36 PCS. Similarly, although others have found poorer outcomes in back pain patients with comorbid depression and anxiety [19,25], we did not find any association between lower mental health scores and lack of improvement.

Limitations of our study include small sample size, use of patient self-reported variables, and lack of long-term follow-up. In addition, the lack of a non-interventional arm in these data precludes determining the component of natural improvement with time. It is possible that we have identified characteristics associated with overall recovery rather the responsiveness to yoga intervention per se. These findings need to be examined further in future studies. Strengths of the study include standard enrollment criteria, outcome measures commonly used in other cLBP trials, and a diverse racial and socioeconomic population.

## Conclusion

Demographic studies show that yoga utilization is highest in white educated women with high socioeconomic status and good health status and less often among minorities, non-English speakers, and individuals with lower incomes and poor health status [5,25]. These different patterns in use are likely due to factors related to access to yoga (e.g., awareness of yoga, availability of yoga instruction, cost of instruction). With the notable exception of education, our results suggest that when yoga is made available to diverse low-income populations with poor health and cLBP, age, race, income, and employment characteristics do not negatively or positively impact the potential to receive benefit.

## Figures and Tables

**Table 1:** Baseline characteristics of 95 adults with chronic low back pain*

Characteristic	

Age	N (%)

<41	25 (26)
41–48	21 (22)
48–55	26 (27)
≥55	23 (24)

Race	

Black	52 (55)

White	17 (18)

Other	26 (27)

Hispanic	9 (9)

U.S. Born	75 (79)

Language spoken at home	
English	81 (85)
Other	14 (15)

Insurance	
Public	53 (56)
Private	41 (44)
None	1 (1)

Education	
Some high school	9 (9)
High school graduate	24 (25)
Some college	21 (22)
College graduate	29 (31)
Graduate school	12 (13)

Income	
≤ $30,000	57 (60)
$30–70,000	24 (25)
> $70,000	9 (9)
Declined	5 (5)

Employed	42 (44)

Pain Duration >1 year	72 (23)

Any medication use in the last week	69 (73)

Sciatica	33 (35)

Satisfied with previous back pain care	17 (19)

Previous yoga use	12 (12)

Any previous CAM use	51 (53)

Note: due to rounding, not all percentages total 100%. See Saper et. al. for additional/prior published population description^11^

**Table 2:** Bivariate analysis of baseline categorical variables and change in primary outcome measures for 91 participants* enrolled in a 12-week yoga trial for chronic low back pain

Characteristic	ΔRMDQ	p-value	ΔLBPS	p-value

Age				

<41	−4.9	0.42	−2.4	0.86
41–48	−4.3		−2.1	
48–55	−4.5		−2.3	
≥55	−6.3		−2.2	

Race				

Black	−4.6	0.42	−2.1	0.61

White	−5.0		−2.7	

Other	−7.0		−2.5	

Hispanic				
Hispanic	−7.3	0.24	−4.0	0.15
Non-hispanic	−4.8		−2.1	

U.S. Born				
U.S. born	−4.1	0.003	−2.1	0.24
Foreign born	−8.9		−3.1	

Language spoken at home				
English	−4.6	0.11	−2.2	0.69
Other	−7.5		−2.6	

Insurance				
Public	−5.3	0.51	−2.5	0.29
Private	−4.4		−1.0	

Education				
Some high school	−4.5	0.55	−0.9	0.027
High school graduate	−4.8		−1.8	
Some college	−4.4		−2.2	
College graduate	−5.4		−2.9	
Graduate school	−5.8		−2.6	

Income categorized				
≤ $30,000	−4.8	0.73	−2.0	0.63
$30–70,000	−6.7		−3.0	
> $70,000	−4.9		−2.3	

Unemployment				
Employed	−5.4	0.67	−2.8	0.18
Not employed	−4.8		−2.0	

Pain Duration				
Pain >1yr	−5.0	0.89	−2.0	0.12
Pain ≤1yr	−4.8		−2.2	

Any medication use in the last week				
Yes	−5.1	0.82	−2.2	0.71
No	−4.8		−2.4	

Over the counter medication use				
Yes	−5.3	0.34	−2.4	0.32
No	−3.5		−1.6	

Prescription medication use				
Yes	−5.0	0.93	−2.1	0.57
No	−5.1		−2.4	

Sciatica				
Yes	−5.2	0.85	−2.1	0.59
No	−4.9		−2.4	

Satisfied with previous back pain care				
Satisfied	−4.8	0.79	−2.3	0.98
Unsatisfied	−5.2		−2.3	

**Other LBP Therapies**				

Trigger point injection				
Yes	−5.3	0.85	−1.9	0.62
No	−5.0		−2.3	

Heat/ice use				
Yes	−4.9	0.75	−2.2	0.95
No	−5.4		−2.3	

Physical therapy				
Yes	−4.9	0.74	−2.1	0.40
No	−5.3		−2.5	

Epidural injection				
Yes	−6.0	0.44	−2.2	0.95
No	−4.8		−2.3	

Surgery				
Yes	−7.3	0.33	−1.5	0.45
No	−4.9		−2.3	

Chiropractor				
Yes	−4.2	0.27	−2.2	0.86
No	−5.6		−2.3	

Massage				
Yes	−4.4	0.37	−2.5	0.56
No	−5.5		−2.2	

Osteopathic manipulation				
Yes	−8.3	0.16	−2.5	0.80
No	−4.8		−2.2	

Previous yoga use				
Yes	−3.1	0.22	−2.4	0.83
No	−5.4		−2.2	

Any previous CAM use				
Yes	−4.6	0.47	−2.4	0.52
No	−5.5		−2.1	

* unless otherwise specified

Abbreviations: RMDQ = Roland-Morris disability questionnaire LBPS = Lower back pain score (0–10 scale of pain) LBP = Lower back pain SF-36 = Short form 36 PCS = Physical Health Component Score MCS = Mental Health Component Score CAM = Complementary and alternative medicine

**Table 3:** Bivariate analysis of baseline continuous variables and change in primary outcome measures for 91 participants* enrolled in a 12-week yoga trial for chronic low back pain

Characteristic	Correlation Coefficient (*r*) with ΔRMDQ	p-value	Correlation Coefficient (*r*) with ΔLBPS	p-value
Baseline RMDQ	−0.33	0.001	−0.18	0.10
Baseline LBPS	−0.09	0.42	−0.46	<.001
SF-36 PCS	0.36	<.001	0.34	0.001
SF-36 MCS	−0.12	0.26	−0.04	0.69
BMI (n=90)	−0.15	0.17	−0.03	0.79
Missed days of work (n=89)	0.26	0.015	0.14	0.20
Hours worked in previous week (n=88)	0.02	0.82	0.03	0.75
Days of decreased activity due to LBP in last 4 weeks (n=89)	−0.06	0.55	−0.00	0.99
Hours of pain/day (n=89)	0.12	0.27	0.10	0.38

* unless otherwise specified

Abbreviations: RMDQ = Roland-Morris disability questionnaire LBPS = Lower back pain score (0–10 scale of pain) SF-36 = Short form 36 PCS = Physical Health Component Score MCS = Mental Health Component Score BMI = Body mass index LBP = low back pain

**Table 4:** Linear regression of baseline characteristics and change in primary outcome measures.

	Change in RMDQ			Change in LBPS		
Variable	Greater improvement in:	β (95% CI)	*p*	Greater improvement in:	β (95% CI)	*p*
U.S. vs. foreign born	Foreign born	5.6 (8.6, 2.6)	<.001	Foreign born		0.20
>HS education	n/a		0.50	College education	−1.2 (−0.3, −2.2)	0.01
Missed work	No missed work	2.7 (5.4, −0.1)	0.054	No missed work		0.12
LBP >1 year	n/a		0.58	n/a	1.1 (2.2, 0.0)	0.04
SF-36 PCS score	Worse physical health	0.3 (0.5, 0.1)	0.02	Worse physical health	0.1 (0.2, 0.0)	0.02

## Abbreviations

cLBP: Chronic Lower Back Pain
RMDQ: Modified Roland-Morris Disability Questionnaire
LBPS: Lower Back Pain Score
PCS: SF-36 Physical Component Score

## References

[1] Deyo RA, Weinstein JN (2001). Low back pain. N Engl J Med.

[2] Deyo RA, Mirza SK, Martin BI (2006). Back pain prevalence and visit rates: estimates from US national surveys 2002. Spine.

[3] Licciardone JC (2008). The epidemiology and medical management of low back pain during ambulatory medical care visits in the United States. Osteopath Med Prim Care.

[4] Guo HR, Tanaka S, Halperin WE, Cameron LL (1999). Back pain prevalence in US industry and estimates of lost workdays. Am J Public Health.

[5] Saper RB, Eisenberg DM, Davis RB, Culpepper L, Phillips RS (2004). Prevalence and patterns of adult yoga use in the United States: results of a national survey. Altern Ther Health Med.

[6] Wolsko PM, Eisenberg DM, Davis RB, Kessler R, Phillips RS (2003). Patterns and perceptions of care for treatment of back and neck pain: results of a national survey. Spine.

[7] Barnes PM, Powell-Griner E, McFann K, Nahin RL (2002). Complementary and alternative medicine use among adults: United States 2002. Adv Data.

[8] Barnes PM, Bloom B, Nahin RL (2008). Complementary and alternative medicine use among adults and children: United States, 2007. Natl Health Stat Report.

[9] Tekur P, Singphow C, Nagendra HR, Raghuram N (2008). Effect of short-term intensive yoga program on pain, functional disability and spinal flexibility in chronic low back pain: a randomized control study. J Altern Complement Med.

[10] Saper RB, Sherman KJ, Cullum-Dugan D, Davis RB, Phillips RS (2009). Yoga for chronic low back pain in a predominantly minority population: a pilot randomized controlled trial. Altern Ther Health Med.

[11] Saper RB, Boah AR, Keosaian J, Cerrada C, Weinberg J (2013). Comparing once- vs. twice-weekly yoga classes for chronic low back pain in predominantly low income minorities: a randomized dosing trial. Evid Based Complement Alternat Med.

[12] Ostelo RW, Deyo RA, Stratford P, Waddell G, Croft P (2008). Interpreting change scores for pain and functional status in low back pain: towards international consensus regarding minimal important change. Spine.

[13] Sherman KJ, Wellman RD, Cook AJ, Cherkin DC, Ceballos RM (2013). Mediators of Yoga and Stretching for Chronic Low Back Pain. Evid Based Complement Alternat Med.

[14] Sherman KJ, Cherkin DC, Ichikawa L, Avins AL, Barlow WE (2009). Characteristics of patients with chronic back pain who benefit from acupuncture. BMC Musculoskelet Disord.

[15] Underwood MR, Morton V, Farrin A, UK BEAM Trial Team (2007). Do baseline characteristics predict response to treatment for low back pain? Secondary analysis of the UK BEAM dataset. Rheumatology (Oxford).

[16] McCracken LM, Turk DC (2002). Behavioral and cognitive–behavioral treatment for chronic pain. Spine.

[17] Harkapaa K, Jarvikoski A, Mellin G, Hurri H, Luoma J (1991). Health locus of control beliefs and psychological distress as predictors for treatment outcome in low back pain patients: results of a 3-month follow-up of a controlled intervention study. Pain.

[18] Hazard RG, Bendix A, Fenwick JW (1991). Disability exaggeration as a predictor of functional restoration outcomes for patients with chronic low back pain. Spine.

[19] Polatin PB, Gatchel RJ, Barnes D, Mayer H, Arens C (1989). A psychosociomedical prediction model of responses to treatment by chronically disabled workers with low back pain. Spine.

[20] Tota-Faucette ME, Gil KM, Williams DA, Keefe FJ, Goli V (1993). Predictors of response to pain management treatment: the role of family environment and changes in cognitive processes. Clin J Pain.

[21] Vendrig AA (1999). Prognostic factors and treatment-related changes associated with return to work in the multimodal treatment of chronic back pain. J Behav Med.

[22] Costa Lda C, Maher CG, McAuley JH, Hancock MJ, Herbert RD (2009). Prognosis for patients with chronic low back pain: inception cohort study. BMJ.

[23] Cecchi F, Negrini S, Pasquini G, Paperini A, Conti AA (2012). Predictors of functional outcome in patients with chronic low back pain undergoing back school, individual physiotherapy or spinal manipulation. Eur J Phys Rehabil Med.

[24] Barnes D, Smith D, Gatchel RJ, Mayer TG (1989). Psychosocioeconomic predictors of treatment success/failure in chronic low back pain patients. Spine.

[25] Birdee GS, Legedza AT, Saper RB, Bertisch SM, Eisenberg DM (2008). Characteristics of yoga users: results of a national survey. J Gen Intern Med.

